# Nanomaterials for Surface Enhanced Raman Spectroscopy

**DOI:** 10.3390/nano13030402

**Published:** 2023-01-18

**Authors:** Andrzej Kudelski

**Affiliations:** Faculty of Chemistry, University of Warsaw, Pasteur Str. 1, 02-093 Warsaw, Poland; akudel@chem.uw.edu.pl

For many decades, Raman spectroscopy has been disregarded as an ineffective analytical tool because of the very low efficiency of “normal” Raman scattering (the typical cross-section for Raman scattering is about 11 and 8 orders of magnitude smaller than the typical cross-sections for absorption in ultraviolet and infrared wavelengths, respectively [1]). However, by utilizing special electromagnetic resonators constructed from plasmonic metals or analogous materials, the Raman scattering signal can be magnified (or enhanced) by many orders of magnitude, thereby enabling the observation of high-quality Raman spectra—even of a single molecule [2,3]. This effect is called SERS (surface-enhanced Raman scattering). This Special Issue of *Nanomaterials*, “Nanomaterials for Surface Enhanced Raman Spectroscopy”, covers the recent advances in nanomaterials for SERS spectroscopy, concerning not only their synthesis but also simulations of the obtained local SERS enhancement factors and the applications of new nanomaterials in chemical SERS analysis.

Muneer et al. described the development of a sensitive and recyclable plasmonic SERS substrate by the deposition of flower-shaped gold nanostructures onto nickel foam [4]. In another contribution, nano-assemblies composed of a gold nanorod core and spherical gold nanoparticle satellites with single-stranded DNA oligomer linkers were produced [5]. This strategy enabled the formation of nano-assemblies that only contained satellites at the nanorod tips, i.e., directional anisotropic nano-assemblies, or satellites randomly positioned around the nanorod, i.e., nondirectional nano-assemblies [5]. Employing self-assembly and Langmuir–Blodgett techniques, Zhang et al. prepared a nanofilm composed of monolayer gold nano-rings sandwiched between a silver mirror (the bottom) and silver cover films (the top) [6]. The produced sandwich nanofilms achieved an overall ~eight-fold SERS signals amplification compared to the solely gold nano-ring layer [6]. The theoretical simulations carried out for this system, which were based on the finite-difference time-domain method, showed consistent results with the experimental ones. In another contribution, the authors developed a genetic-algorithm-based optimization method that allows SERS substrates to achieve strong electric field localization over wide areas for reconfigurable and programmable photonic SERS sensors [7]. 

Moldovan et al. described various strategies for the detection of organochlorine pesticides with SERS [8], whereas Krajczewski et al. described the preparation and characterization of substrates for SERS experiments on nanostructured, non-metallic materials [9].

In summary, this Special Issue presents several examples of the latest advancements in the synthesis and application of certain nanomaterials for SERS spectroscopy. I hope the readers will enjoy reading these articles and find them useful for their research.
